# The effects of caregiver's burden on dynamic structure in disorder of consciousness families: An observational study

**DOI:** 10.1002/brb3.2305

**Published:** 2021-08-05

**Authors:** Francesco Corallo, Deborah Pria, Agata Di Blasi, Lilla Bonanno, Maria Cristina De Cola, Marcella Di Cara, Carmela Rifici, Simona De Salvo, Placido Bramanti, Silvia Marino, Viviana Lo Buono

**Affiliations:** ^1^ IRCCS Centro Neurolesi “Bonino‐Pulejo” Messina Italy

**Keywords:** caregivers, disorders of consciousness, minimally conscious state, psychological function

## Abstract

**Introduction:**

Disorder of consciousness is a clinical condition due to severe brain damage. The impact of consciousness disorder on the family is characterized by a combination of biopsychosocial factors. The burden and suffering perceived by caregivers can cause psychological distress characterized by anxiety, depression, and physical illness. The aim of the study was to investigate the interaction between family dynamics and caregiver burden.

**Methods:**

We enlisted 35 caregivers of subjects in a minimally conscious state. Two skilled psychologists administered the Olson's Adaptability and Family Cohesion Assessment Scale and the Novak's Burden Inventory Caregiver Scale to assess family function and family burden, respectively.

**Results:**

We found that the caregiver burden correlates with the family adaptability and cohesion, as well as with enmeshment, rigidity, and disengagement.

**Conclusion:**

Findings suggest that the traumatic event does not affect the family structure. Families are able to maintain a balanced functioning and control distress.

## Graphical abstract

Disorder of consciousness is a clinical condition due to severe brain damage. The burden and suffering perceived by caregivers can cause psychological distress characterized by anxiety, depression and physical illness. The patient and his/her family should be followed by a multidisciplinary team including a neuropsychologist, who can facilitate the family dynamic structure and to safeguard the welfare of the patient.

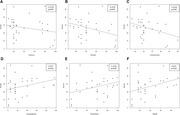

## 1 INTRODUCTION

The disorder of consciousness is a clinical condition where consciousness is affected by severe cerebral damage. It includes two main states: (i) the vegetative state (VS), a condition of unawareness of self or environment with regular sleep–wake cycles and characterized by complete or partial preservation of hypothalamic and brain stem autonomic functions (Kim et al., 2012; Marino et al., 2012); (ii) the minimally conscious state (MCS), where there is minimal behavioral evidence of self‐awareness or environmental awareness (Marino et al., 2013). MCS patients require hospitalization providing intensive medical‐rehabilitative interventions from a few weeks to several months. The care of these patients involves the active participation of the family system and primary caregiver for the duration of the rehabilitation process. Indeed, the caregiver plays an important role in the management of the frail patient (Guarnerio et al., 2012; Marino et al., 2013), which can affect his/her lifestyle and lead to a hierarchical rearrangement. Caregivers often lose interest in their hobbies and dedicate less time to work and themselves. Sometimes, the condition is so stressful that it may lead to the abandonment of work (Corallo, Bonanno, De Salvo, et al., 2015; Sherwood et al., 2008). In some cases, family members are not able to accept the condition of their loved ones and may experience psychosomatic disturbances, insomnia, loss of appetite, anxiety, anger, and aggressiveness (Chiambretto et al., 2001; Ho et al., 2009; Jacobs et al., 1986). According to a biopsychosocial theory, it is important to consider the needs of caregivers because they are a resource for patient care. Indeed, the burden due to care of patients with chronic illness involves physical, psychological, emotional, social, and financial aspects. Moreover, it is often characterized by anxiety, depression, and physical illness (Bayen et al., 2013; Leggett et al., 2010; Leonardi et al., 2012).

In this scenario, it becomes necessary to adopt a new clinical approach, systemic, that allows to understand how a family can modulate stress in critical moments through its interior dynamics. The way each family handles a crisis affects the adaptation processes, and therefore, the psychosocial well‐being of all family members.

Family functioning refers to the social and structural quality of the family environment, including conflict and cohesion among family members, and also adaptability, organization, and communication. The family functioning aims to integrate various characteristics of the family, consider the family as a system, and examine the overall functioning of such a system. Thus, each member contributes to the family functioning, and after a traumatic event that led to an MCS condition in a family member, the whole family goes through different phases (Cigoli & Mariotti, 2002; Leibach et al., 2014), from denial to reorganization, up to the acceptance of the situation and the creation of a new equilibrium (Lezak 1988; Rolland & Walsh, 2005; Tzidkiahu et al., 1994). Therefore, the event as the clinical condition of the patient as an MCS can affect family balances through the combination of biopsychosocial factors, arising also in care management.

The purpose of this study was to investigate adaptability and cohesion of the families in a sample of primary caregivers of MCS patients, assessing the possible correlation between family functioning and the caregiver burden.

## 2 METHODS

This is a cross‐sectional study. We consecutively enrolled 50 primary caregivers of patients being in an MCS. Participants were recruited within one month from the hospitalization at the Rehabilitation Unit for acquired severe brain injury patients of the IRCCS Centro Neurolesi “Bonino Pulejo'' of Messina, where patients received rehabilitation by a multidisciplinary team consisting of several health professionals from June 2017 to February 2018. Due to the lack of data, 15 participants were excluded and our final sample included only 35 caregivers. A more detailed description of the sample is provided in Table 1.

**TABLE 1:** Sociodemographic characteristics and clinical scores of the sample group

Socio‐demographic characteristics	
Age (Mean ± SD) (years)	56.08 ± 10.96
Gender female	24 (68.6)
Education	
Elementary school	8 (22.9)
Middle school	10 (28.6)
High school	15 (42.8)
University	2 (5.7)
Marital status	
Single	4 (11.4)
Married/living with partner	28 (80.0)
Separated/divorced	3 (8.6)
Relationship with the patient	
Father/mother	7 (20.0)
Son/daughter	9 (25.7)
Spouse/partner	17 (48.6)
Other	2 (5.7)
Cohabitation with the patient	31 (88.6)
Clinical scores	
Caregiver burden inventory	28.0 (20.0–34.0)
FACES‐IV	
Cohesion	25.0 (20.0–72.5)
Flexibility	30.0 (25.0–52.5)
Disengagement	15.0 (5.0–40.0)
Enmeshment	65.0 (45.0–80.0)
Rigidity	30.0 (17.0–55.0)
Disorganization	1.0 (1.0–28.0)
Communication	30.0 (28.0–34.0)
Satisfaction	31.0 (30.0–36.0)

*Note*: Median (I‐III quartile) were used to describe continuous variables; frequencies (percentages) were used to describe categorical variables.

All participants were literate in Italian language and consented to participate in the study by signing a written consent form. The Local Ethics Committee approved the study protocol according to the Declaration of Helsinki.

### 2.1 Assessment

Two skilled psychologists administered the Italian version of the two following instruments: Family Adaptability and Cohesion Evaluation Scale (FACES‐IV) by Olson and Caregiver Burden Inventory (CBI) by Novak (1989).

The FACES‐IV (Visani et al., 2004) is a self‐report interview measuring the family aspect assessment in terms of cohesion and adaptability in order to identify the facilitating dimension of a functional family and the feeling of closeness and attachment of family members to each other. The used version included six scales (two balanced and four unbalanced) of seven items each, making 42 items, according to Circumplex Model of Olson, that is, a circumplex ratio score ranging from 0 (worst) to 10 (best). A score ⩾1 indicates balanced levels of cohesion and flexibility in the system. In addition, 10 items assess the level of family communication with the Family Communication Scale, and 10 items assess the level of family satisfaction along the Family Satisfaction Scale. Thus, the interview had a total of 62 items. The FACES‐IV recognizes six types of families: cohesive, flexible, disengaged, entangled, rigid, and chaotic. A healthy family environment is characterized by clear communication, well‐defined roles, enmeshment, and cohesion among family members. On the contrary, poor family functioning occurs within families with high levels of disorganization, rigidity, disengagement, and behavioral control.

The CBI scale is a self‐report interview measuring the burden of caregivers. It is characterized by 24 items on five dimensions: time‐dependence burden, developmental burden, physical burden, social burden, and emotional burden. A total score >36 indicates a risk of “burning out,” whereas scores near or slightly above 24 indicate a need to seek some form of respite care.

### 2.2 Statistical analysis

Continuous variables were expressed as mean ± SD or median (I‐III quartile) as appropriate. Categorical variables were expressed as frequencies and percentages. A nonparametric analysis was carried out because the results of the Shapiro normality test indicated that most of the target variables were not normally distributed. Thus, correlations among variables were computed by Spearman's coefficient, and statistical differences were assessed by Mann–Whitney *U* test in continuous variables or Chi‐square test in proportions.

In order to investigate the influence of burden on the family functioning, we performed a series of multiple regression analysis by fixing the FACES‐IV subitem scores as dependent variables and CBI score as predictor, controlling for demographics (age, gender, education, and marital status). We applied a backward elimination stepwise procedure for the choice of the best predictive variables according to the Akaike information criterion (AIC).

A formal power analysis was not performed in advance. Thus, this study should be considered as explorative, being not able to detect smaller yet clinically relevant differences. Analyses were performed using the open source R3.0 software package. Statistical significance was set at *p* < .05.

## 3 RESULTS

### 3.1 Descriptive analysis

As shown in Table 1, around 69% of participants were female, 31–72 years aged (median age of 55 years), and with a medium level of education. About 80% were married or lived with a partner, and 89% lived with an MCS patient. Notably, almost the whole sample (94%) included a direct family member caring for an MCS patient. No significant differences in CBI scores between women and men emerged, as well as when we grouped by marital status. We found a significant association between the relationship with the patient and the marital status (*χ* ^2^(6) = 15.90, *p* = .01) and the cohabitation with him/her (*χ* ^2^(3) = 17.75, *p* < .001). Indeed, about half of the samples (48.6%) were the spouses of the patients.

Concerning the perception of the family dimensions, we found low levels of cohesion, and medium level of flexibility, as reported in Table 1.

### 3.2 Spearman correlation analysis

**FIGURE 1:**
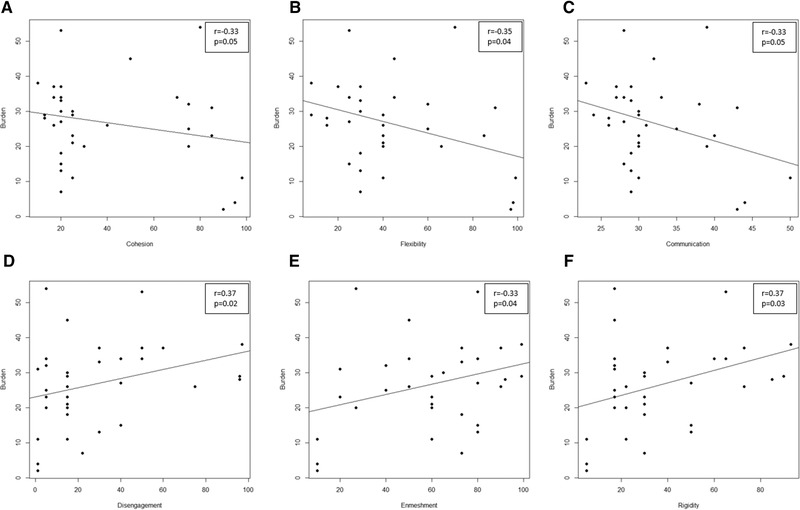
Significance correlation between burden and subitem of Family Adaptability and Cohesion Evaluation Scale (FACES‐IV) scores. (a) Scatter plot of burden scores and cohesion. (b) Scatter plot of Burden scores and Flexibility. (c) Scatter plot of burden scores and communication. (d). Scatter plot of burden scores and disengagement. (e) Scatter plot of burden scores and enmeshment. (f) Scatter plot of burden scores and rigidity

As shown in Figure 1, many moderate correlations between CBI scores and the FACES‐IV subitem scores emerged. In particular, we observed a negative correlation between the burden and flexibility (*r* = –0.35; *p* = .04), as well as positive correlations between the burden and disengagement (*r* = 0.37; *p* = .02), enmeshment (*r* = 0.33; *p* = .04), and rigidity (*r* = 0.37; *p* = .03).

### 3.3 Multiple regression analysis

Results in Table 2 showed that the burden was a significant predictor for three FACES‐IV subscales: flexibility, enmeshment, and rigidity.

**TABLE 2:** Multiple regression: Significant predictors on each subitem of Family Adaptability and Cohesion Evaluation Scale (FACES‐IV) scores

Dependent variables	Predictors	*β*	Std *β*	*p*‐value	Adjusted *R* ^2^
Flexibility	Burden	–0.77	–0.36	.04	0.05
Enmeshment	Burden	0.72	0.33	.04	0.04
Rigidity	Burden	0.80	0.38	.02	0.10

*β* = regression coefficient; Std *β* = standardized regression coefficient.

## 4 DISCUSSION

In this study, we investigated how different family characteristics could influence the way to deal with the MCS situation of a family member. Contrary to our hypothesis, this study showed good levels of cohesion and flexibility correlated with a high level of burden in the family structures. This suggests a facilitating in management within the family. The correlation between high levels of enmeshment and low burden suggests that the more the family is involved, the more difficult is to manage the burden. However, standard values of communication and satisfaction, together with low levels of disorganization, lead us to consider a balanced functioning within the family structure, reflecting the member's ability to listen, respect, and attention.

Few studies examine aspects of family functioning in brain injured patients, especially during the rehabilitative training (Maggio et al., 2018; Kreutzer et al., 2016), although many studies concern the burden of caregivers and the benefit due to suitable psychological training. For instance, Corallo et al. (2018) studied the changes that occur in the caregivers after psychological support provided during the hospitalization of the patient at the postacute rehabilitation unit.

Previous studies showed that family members could develop fantasies about the patient's state of awareness, interpreting spastic movements or reflexes as improvement signs (Tzidkiahu et al., 1994). It has been observed that the protracted assistance causes caregivers psychosomatic disorder, insomnia, and lack of appetite. Leonardi et al. (2018) evaluated the caregiver burden of disorder of consciousness patients and its impact on caregivers’ life. They described an objective dimension of burden that includes realistic changes in personal life, as well as subjective dimension, arguing that it is impossible to distinguish interpersonal level, such as self‐perception in relation to the environment or interpersonal relations with the patients, from intrapersonal level with anxiety and depression symptoms, general mental health, and others. Soeterik et al. (2018) analyzed the reaction of a sample of women caring for relatives with disorder of consciousness, observing that the condition created uncertainty in their lives and their future as a wife and/or mother.

In another previous study, Corallo, Bonanno, Lo Buono, et al. (2015) described aspects affecting the crisis of the family system as the unpredictability of the accident, the risk for death of the relative and efforts to accept his/her behavioral disorders, frustrations related to the long recovery times, and last but not least, financial difficulties. In contrast, the families included in our sample reflected balanced functioning, able to adapt to new events by balancing extreme behaviors, and independence and connection between family members. Moreover, our findings showed that the caregiver's burden was negatively correlated with rigidity and disengagement. This could be probably due to the long time spent with patients, which restricted the time for themselves.

The main limitations of the study are the small sample size and its cross‐sectional design. Indeed, the small sample size may not enable us to generalize our findings, whereas a longitudinal study could allow a clear assessment of any temporal relationships between caregiver's burden and family dynamic to justify the inferences, for example, it might be that the level of burden improves over time in relation to disease duration or length of the hospitalization. Similarly, with a control group it could determine the effectiveness of a family‐centred intervention specific for caregivers. However, the most recent literature supports our findings also in a two‐armed randomized controlled trial (Rasmussen et al., 2021). Further large‐scale studies should be carried out to confirm or contradict our findings and hypothesis, for example, investigating differences due to specific characteristics as the family relation, the etiology or the side of injury, the level of cognitive impairment, and the length of the hospitalization.

## 5 CONCLUSION

Moderate correlations of cohesion and flexibility with level of burden suggest that the traumatic event does not affect the family structure. Family members are able to maintain the roles within the family system, controlling distress.

## CONFLICT OF INTEREST

The authors declare no conflict of interest.

## Data Availability

Data are not available for this study given that account holders could be identifiable if it were made accessible.
